# Supplementary material for: Sustained viremia suppression by SHIVSF162P3CN-recalled effector-memory CD8+ T cells after PD1-based vaccination
Source: PLoS Pathog. 2021 Jun 14;17(6):e1009647. doi: 10.1371/journal.ppat.1009647 (PMC8202916; doi:10.1371/journal.ppat.1009647)
Supplement: S1 Table — MHC class-I genotypes was determined by deep sequencing to determine whether vaccinated macaques carried protective MHC class I allele. (DOCX) [file ppat.1009647.s001.docx]

**S1 Table**

**MHC class I genotyping of the vaccinated macaques**

| **Macaque #** | **MHC *Mamu* class I alleles** |
| --- | --- |
| A01 | A1*026, A1*051  B*007, B*013, B*030, B*068, B*070, B*074, B*082 |
| A02^^^ | A1*003/A1*019, A1*040, A4*014,  B*015, **B*017**, B*036, B*037, B*045, B*050, B*068, B*078, B*167 |
| A03 | A1*049, A1*051, A2*005, A3*013/A4*003, A4*014  B*010, B*041, B*048, B*068, B*087 |
| A04^^^ | A4*14, A1*026, A1*028  B*001, B*007, **B*017**, B*030, B*065, B*083 |
| C01 | A1*048/A1*120, A2*024  B*030, B*068, B*069, B*072, B*082 |
| C02 | A1*003, A1*004, A1*048/A1*120  B*013, B*030, B*065, B*068 |
| C03 | A1*022, A1*026  B*030, B*044, B*050, B*068, B*074, B*086, B*151 |

^ The bold and underlined Mamu-B*017 allele in macaques A02 and A04 is known to be associated with better SIV control [23,24­].
