# Supplementary material for: Sustained viremia suppression by SHIVSF162P3CN-recalled effector-memory CD8+ T cells after PD1-based vaccination
Source: PLoS Pathog. 2021 Jun 14;17(6):e1009647. doi: 10.1371/journal.ppat.1009647 (PMC8202916; doi:10.1371/journal.ppat.1009647)
Supplement: S2 Table — Single-cell RNA sequencing was done for Group B macaques before last immunisation, 4 weeks post, and 12 weeks post last immunisation using PBMCs. Data analysis was done as described under Materials and Methods. (DOCX) [file ppat.1009647.s002.docx]

| **S2 Table**  **Top 20 differentially expressed genes within each CD8^+^T cell clusters** | | | | | | | |
| --- | --- | --- | --- | --- | --- | --- | --- |
| **CD8^+^T cell cluster** | | | | | | | |
| **1** | **2** | **3** | **4** | **5** | **6** | **7** | **8** |
| SQSTM1 | LTB | SELL | CAPG | TYROBP | GZMB | GZMK | ENSMMUG00000013779 |
| ENSMMUG00000042180 | IGFBP2 | CA6 | SPINK2 | TRBV28 | ENSMMUG00000039070 | IL7R | GNLY |
| HIST1H2AC | TCF7 | CCR7 | IL7R | KLRC3 | GZMH | SPINK2 | TYROBP |
| ENSMMUG00000002162 | TIMP1 | LEF1 | ITGB7 | GNLY | CX3CR1 | CXCR3 | KLRC3 |
| TAF1D | CPE | ENSMMUG00000020989 | ITGB1 | ENSMMUG00000042057 | CST7 | GZMM | ENSMMUG00000042057 |
| ENSMMUG00000028703 | MFGE8 | PLAC8 | VIM | GZMA | NKG7 | VIM | SPINK2 |
| SPOCK2 | MTSS1 | RGS10 | EGLN3 | CCL5 | MAMU-DPB | PVRIG | MAMU-KIR |
| ENSMMUG00000028701 | HMGN3 | SPOCK2 | TNFSF13B | ENSMMUG00000038528 | CD52 | COTL1 | ENSMMUG00000020102 |
| SLC3A2 | ENSMMUG00000029772 | TCF7 | GPR183 | ENSMMUG00000042343 | SH3BGRL3 | ITM2C | GZMM |
| ENSMMUG00000049151 | LAT2 | LTB | S100A6 | FCGR3 | S100A4 | MT1E | LGALS1 |
| RF00100.101 | ID3 | PECAM1 | ODAPH | ENSMMUG00000018327 | APOBEC3C | ERN1 | CTSW |
| LEF1 | ENSMMUG00000013779 | ENSMMUG00000029772 | SPOCK2 | MAMU-KIR | S100A10 | MT1M | ENSMMUG00000023454 |
| CCR7 | ITM2C | TMIGD2 | CLDND1 | GZMB | AHNAK | EIF4EBP1 | IL2RB |
| UBE2B | ZNF683 | RCAN3 | DPEP2 | RHOC | KLRF1 | CYB5A | LDHA |
| HSPA5 | ENSMMUG00000042057 | ITGA6 | PDCD4 | OPLAH | ITGB2 | APOBEC3H | KLRB1 |
| CARS | ENSMMUG00000043342 | FCMR | ANXA2 | AFAP1L2 | SH3BP5 | COA1 | ENSMMUG00000012140 |
| PTP4A1 | IL7R | HPCAL1 | LTB | ENSMMUG00000020102 | TGFBR3 | CD96 | SERPINB6 |
| PPP1R2 | KLRB1 | SATB1 | TNFSF10 | GZMH | FLNA | CCDC167 | S100A6 |
| H2AFZ | CXCR3 | ACTN1 | IL4R | KLRB1 | ID2 | CLDND1 | ENSMMUG00000029875 |
| RPS27L | CTSW | RFLNB | TNFRSF25 | LITAF | CCL5 | KCNA3 | CXCR3 |
| The above listed the top 20 differentially expressed genes of each CD8^+^T cell clusters, and genes with the highest expression were listed from the top to bottom | | | | | | | |
