# Supplementary material for: Sustained viremia suppression by SHIVSF162P3CN-recalled effector-memory CD8+ T cells after PD1-based vaccination
Source: PLoS Pathog. 2021 Jun 14;17(6):e1009647. doi: 10.1371/journal.ppat.1009647 (PMC8202916; doi:10.1371/journal.ppat.1009647)
Supplement: S3 Table — Differentially expressed genes were annotated by GO analysis as described under Materials and Methods. (DOCX) [file ppat.1009647.s003.docx]

| **S3 Table**  **Differentially expressed genes under the top 3 immunology related pathways in Cluster 5, 6, and 8 annotated by GO analysis** | | | | | | | | | | | | | | | | |
| --- | --- | --- | --- | --- | --- | --- | --- | --- | --- | --- | --- | --- | --- | --- | --- | --- |
| **Immune Response** | | | | | | | | | | | | | | | | |
| **Cluster** | ACTR3 | ANXA1 | APOBEC3G | BST2 | CCL5 | CD59 | CD74 | CST7 | FCGR3 | GAPDH | GNLY | HCST | ITK | KLRD1 | LAT2 | LCP2 |
| **5** | ✓ | ✓ | ✓ | ✓ | ✓ | ✓ | ✓ | ✓ | ✓ |  | ✓ | ✓ |  |  | ✓ |  |
| **6** | ✓ | ✓ | ✓ |  | ✓ |  | ✓ | ✓ |  | ✓ |  | ✓ | ✓ | ✓ |  | ✓ |
| **8** |  |  |  | ✓ |  | ✓ | ✓ |  |  |  |  | ✓ |  |  |  |  |
| **Cluster** | MAMU-A | MAMU-A3 | MAMU-B | MAMU-DPA | MAMU-DPB | MAMU-DRA | MAMU-DRB1 | MAPK1 | PTPN22 | PTPN6 | PYCARD | SAMHD1 | SH2D1A | TYROBP | ZAP70 | ZYX |
| **5** |  |  |  | ✓ |  | ✓ |  | ✓ | ✓ | ✓ | ✓ |  |  | ✓ |  | ✓ |
| **6** | ✓ | ✓ | ✓ | ✓ | ✓ | ✓ | ✓ |  |  |  |  |  | ✓ |  | ✓ | ✓ |
| **8** |  |  |  |  |  |  |  |  | ✓ |  |  | ✓ |  |  |  |  |
| **Regulation of Immune System Process** | | | | | | | | | | | | | | | | |
| **Cluster** | ANXA1 | BST2 | CCL5 | CD59 | CD74 | CXCR3 | FCGR3 | HCST | LAT2 | MAPK1 | PTPN22 | PTPN6 | PYCARD | SAMHD1 | TESC | TYROBP |
| **5** | ✓ | ✓ | ✓ | ✓ | ✓ |  | ✓ | ✓ | ✓ | ✓ | ✓ | ✓ | ✓ |  | ✓ | ✓ |
| **8** |  | ✓ |  | ✓ | ✓ | ✓ |  | ✓ |  |  | ✓ |  |  | ✓ |  | ✓ |
| **Regulation of Immune Response** | | | | | | | | | | | | | | | | |
| **Cluster** | ANXA1 | BST2 | CD59 | CD74 | FCGR3 | HCST | LAT2 | MAPK1 | PTPN22 | PTPN6 | PYCARD | SAMHD1 | TYROBP |  |  |  |
| **5** | ✓ | ✓ | ✓ | ✓ | ✓ | ✓ | ✓ | ✓ | ✓ | ✓ | ✓ |  |  |  |  |  |
| **8** |  | ✓ | ✓ | ✓ |  |  |  |  |  |  |  | ✓ | ✓ |  |  |  |
| ✓ represents differential gene expressed under the pathway  No differentially expressed genes were annotated under "Regulation of Immune System Process" and "Regulation of Immune Response" for Cluster 6 | | | | | | | | | | | | | | | | |
