# Supplementary material for: Sustained viremia suppression by SHIVSF162P3CN-recalled effector-memory CD8+ T cells after PD1-based vaccination
Source: PLoS Pathog. 2021 Jun 14;17(6):e1009647. doi: 10.1371/journal.ppat.1009647 (PMC8202916; doi:10.1371/journal.ppat.1009647)
Supplement: S4 Table — Animal ID assigned in this study and its corresponding ID in the breeding animal facility or animal facility for animal experiments were shown. (DOCX) [file ppat.1009647.s004.docx]

**S4 Table**

**Corresponding identification numbers of the tested animals from the animal facility**

| Animal ID assigned in this study | Corresponding ID in the breeding animal facility | Corresponding ID in the animal facility |
| --- | --- | --- |
| A01 | 11-2480R | 150705 |
| A02 | 11-2646R | 150706 |
| A03 | 11-2474R | 150707 |
| A04 | 12-1596R | 150708 |
| B01 | 12-1450R | 150701 |
| B02 | 12-1448R | 160306 |
| B03 | 12-1342R | 160307 |
| C01 | 11-2312R | 150702 |
| C02 | 12-1304R | 160308 |
| C03 | 12-1318R | 160309 |
| C04 | 13-2310R | 171001 |
| C05 | 13-2360R | 171002 |
| C06 | 13-2210R | 171003 |
| C07 | 13-2344R | 171011 |
| C08 | 13-1784R | 171012 |
| C09 | 14-1474R | 180505 |
