# Supplementary figures and images for: Sustained viremia suppression by SHIVSF162P3CN-recalled effector-memory CD8+ T cells after PD1-based vaccination
Source: PLoS Pathog. 2021 Jun 14;17(6):e1009647. doi: 10.1371/journal.ppat.1009647 (PMC8202916; doi:10.1371/journal.ppat.1009647)

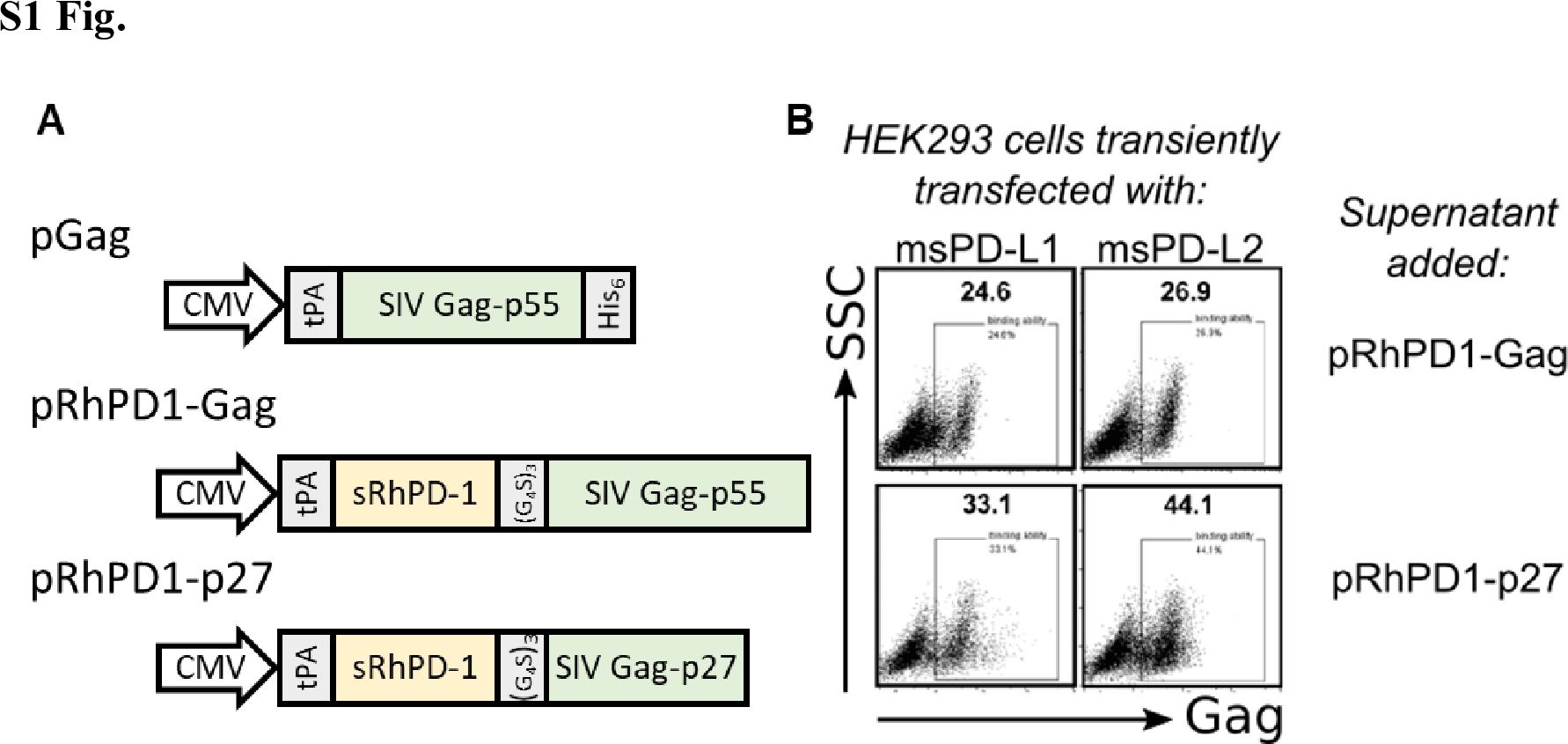

Supplement: S1 Fig — (A) Three DNA vaccine candidates were constructed using pVAX as the expression vector. A pair of DNA vaccines, expressing SIVmac Gag-p55 antigen, either alone (pGag) or fused to rhesus soluble PD1 domain (pRhPD1-Gag), were generated. DNA vaccine, pRhPD1-p27, was also constructed to encode for a rhesus soluble PD1 domain fused to the Gag-p27 capsid antigen. A (G4S)3 linker sequence was placed in between soluble PD1 domain and the antigen in pRhPD1-Gag and pRhPD1-p27. All Gag antigens were placed under the CMV promoter and contained a human tissue plasminogen activator (tPA) secretory signal sequence to promote antigen secretion. All constructs were codon optimised for expression in mammalian cells. (B) Soluble proteins pRhPD1-Gag and pRhPD1-p27 expressed and released from transfected HEK293 cells were confirmed for binding to msPD-L1 and msPD-L2, respectively. (TIF) [file ppat.1009647.s005.tif]

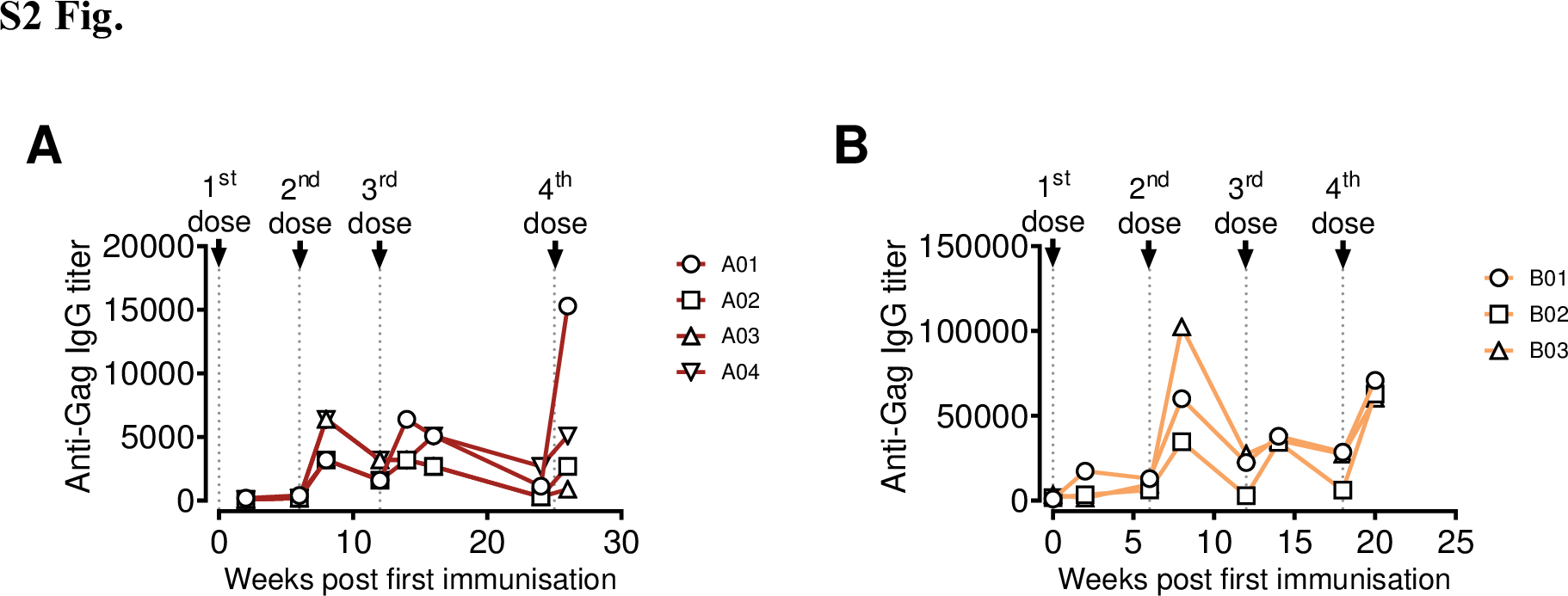

Supplement: S2 Fig — Anti-Gag IgG antibody dilution titers were measured by ELISA in the plasma isolated from pRhPD1-p27-vaccinated rhesus macaques from Group A (A) and Group B (B) at time points indicated. (TIF) [file ppat.1009647.s006.tif]

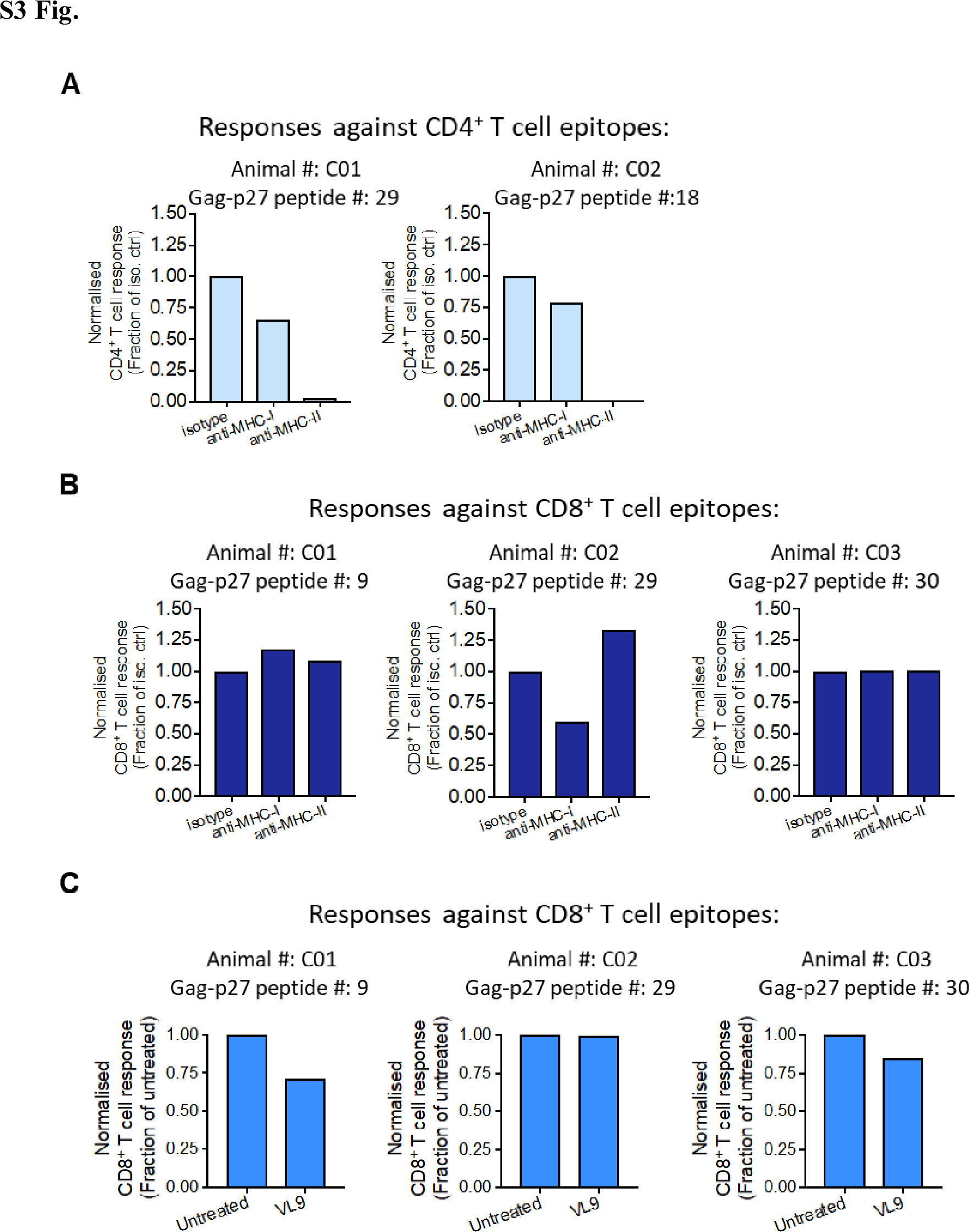

Supplement: S3 Fig — PBMCs isolated at 10 weeks (A and B) or at 8 weeks (C) post-last immunization from the immunized macaques were firstly incubated with anti-MHC-I, anti-MHC-II antibodies, or MHC-E-blocking VL9 peptide for 2 hours at 37°C with 5% CO2. Individual Gag-p27 peptides corresponding to the mapped T cell epitopes were then added to the cells and incubated at 37°C with 5% CO2. 2 hours later, BFA was added two hours later. After overnight incubation, cells were washed and stained for surface markers, followed by fixation with 2% PFA and stained for TNF-α and IFN-γ in Perm/Wash buffer. T cell responses, as determined by TNF-α+ and IFN-γ+, were determined using FACS. Results were normalized against the non-blocking isotype or untreated controls. (TIF) [file ppat.1009647.s007.tif]

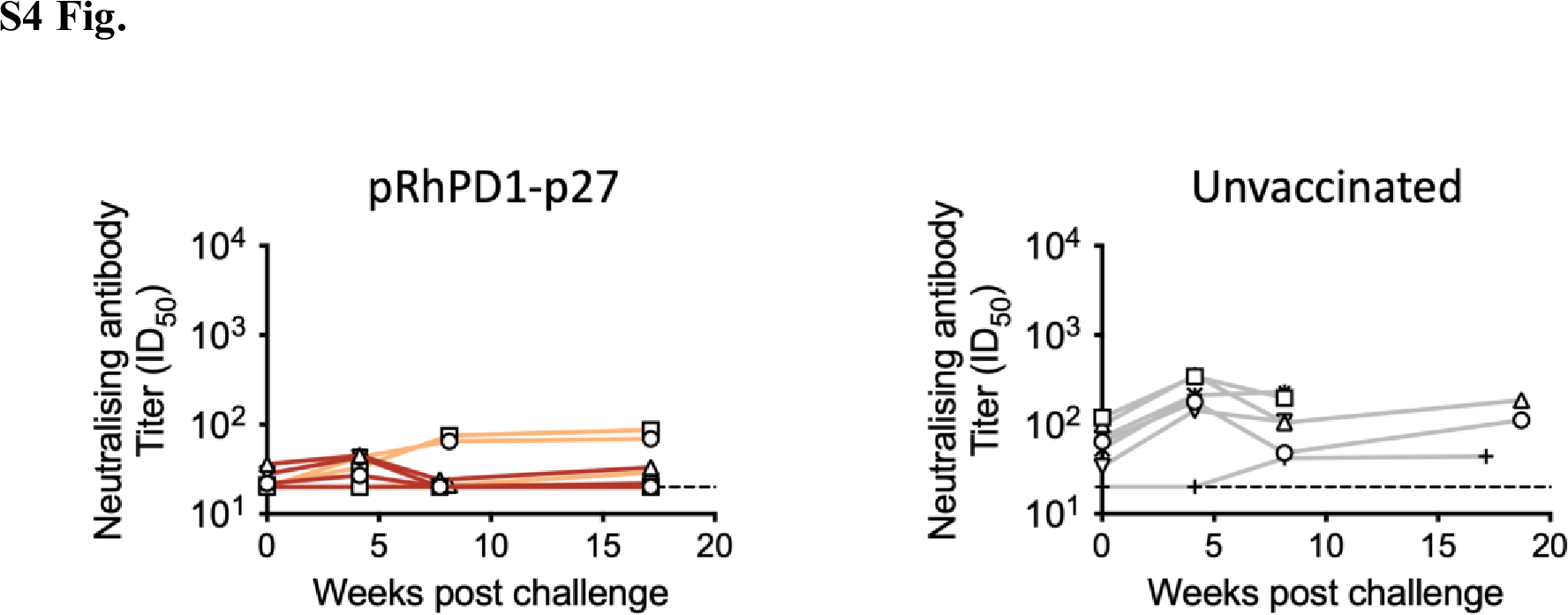

Supplement: S4 Fig — Plasma from the infected macaques was samples in the indicated timepoint to test the neutralizing antibody activity against SHIVSF162P3CN with TZM-bl cells using luciferase reporter assay. (TIF) [file ppat.1009647.s008.tif]

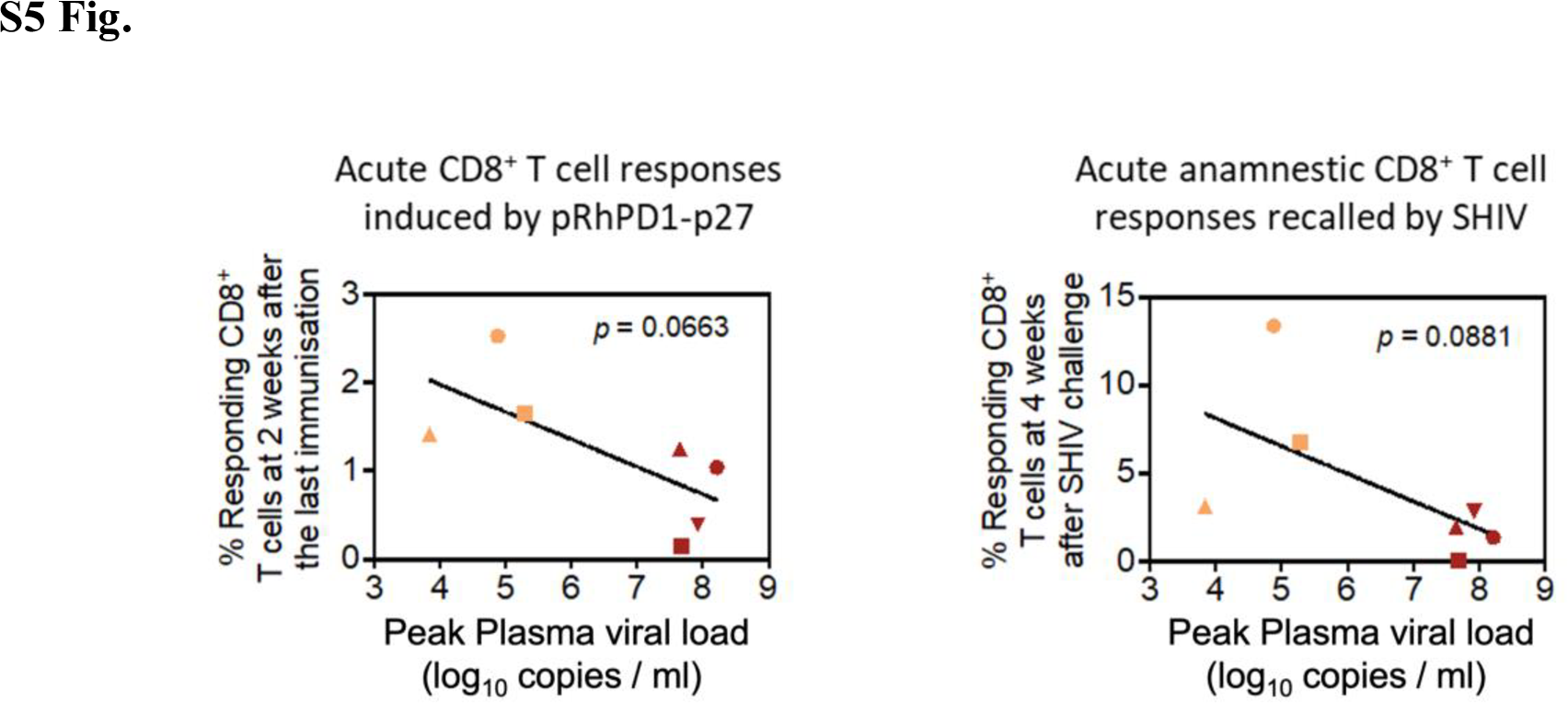

Supplement: S5 Fig — Vaccinated macaques from both groups A and B are shown in this analysis. p valves shown were calculated based on the Spearman rank-correlation test. (TIF) [file ppat.1009647.s009.tif]

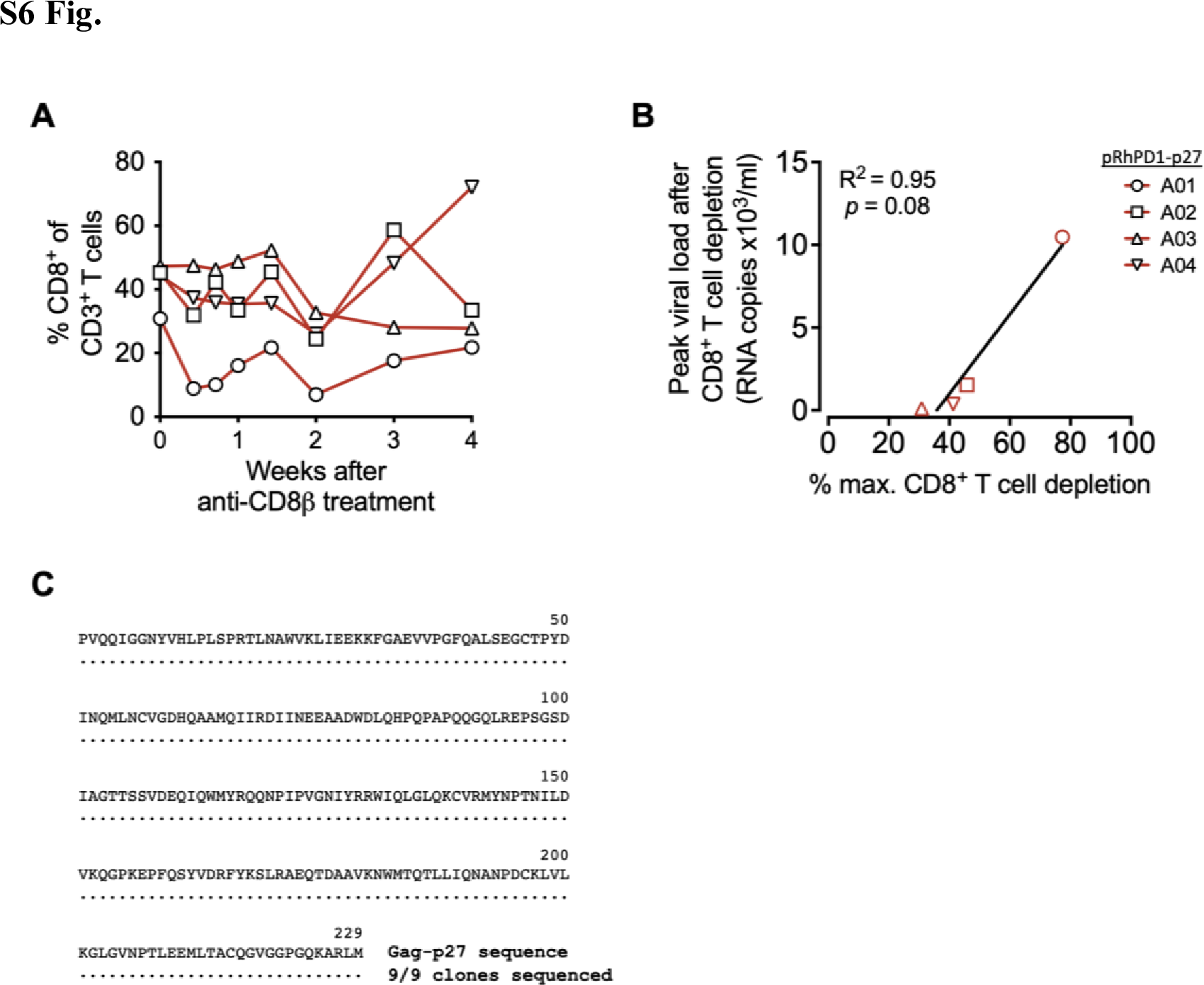

Supplement: S6 Fig — (A) Changes of peripheral CD8+ T cell frequency after intravenous injection of anti-CD8β depleting antibody CD8b255R1. (B) Correlation of peak viral load after CD8+ T cell depletion and the magnitude of CD8+ T cell depletion. p valves shown were calculated based on the Spearman rank-correlation test. (C) Sequence analysis did not show escape mutations in Gag-p27 encoded in the vaccine during viral load rebound. (TIF) [file ppat.1009647.s010.tif]

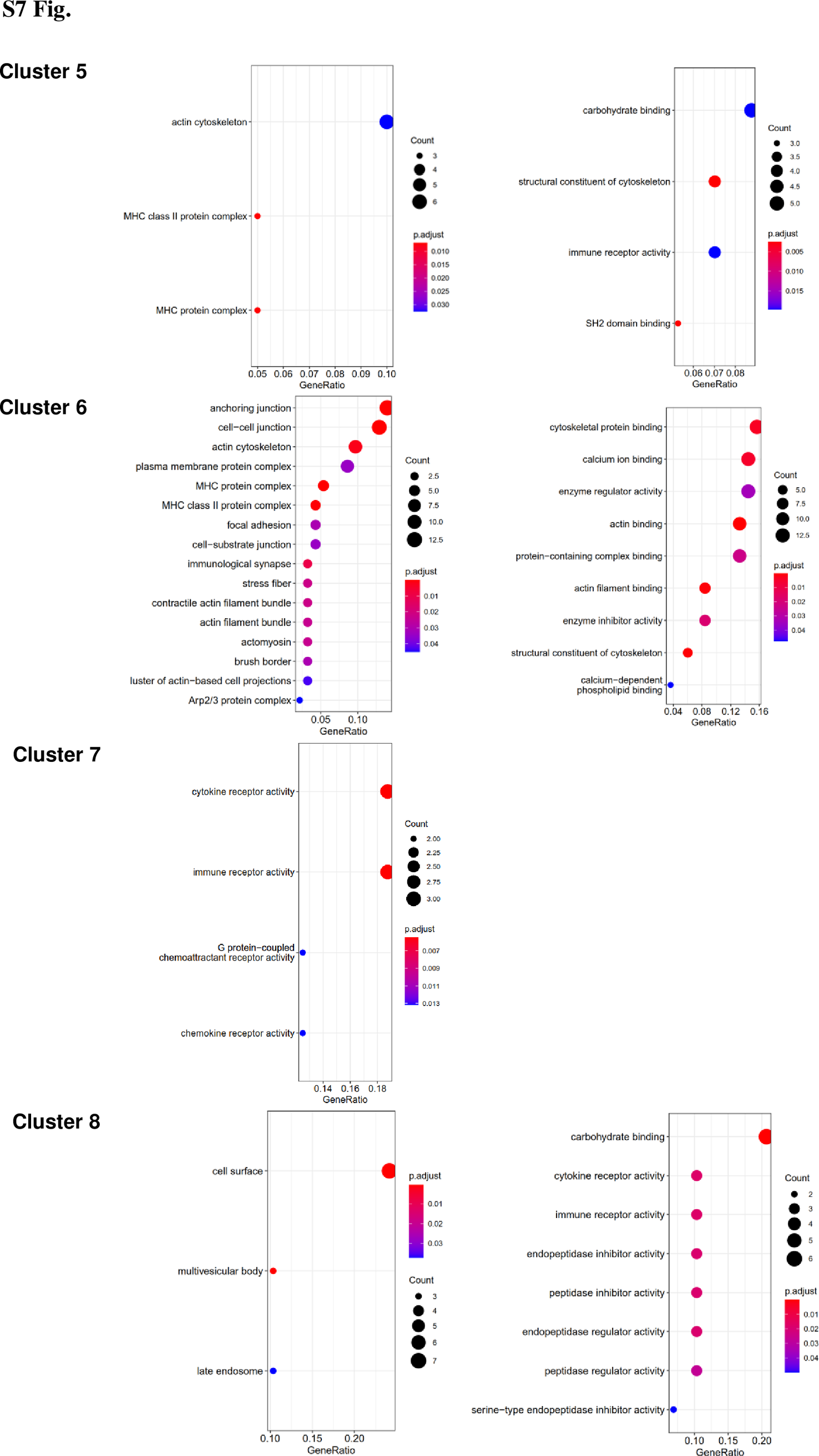

Supplement: S7 Fig — GeneRatio represents the ratio of the number of genes related to the GO term to the total number of significant genes. For Cluster 7, no DEGs were enriched in pathways related to cellular component. (TIF) [file ppat.1009647.s011.tif]
