# Supplementary material for: Sustained viremia suppression by SHIVSF162P3CN-recalled effector-memory CD8+ T cells after PD1-based vaccination
Source: PLoS Pathog. 2021 Jun 14;17(6):e1009647. doi: 10.1371/journal.ppat.1009647 (PMC8202916; doi:10.1371/journal.ppat.1009647)
Supplement: Attachment — Submitted filename: Wong et al response to reviewers.docx [file ppat.1009647.s015.docx]

We thank both reviews for their supportive comments and suggestions. The author summary is included as requested and other changes are highlighted in yellow. The corresponding clean version is also submitted together with this reply. Our specific responses are as follows.

**Part I - Summary**

1) *Comment: Fig 2 – the numbers are small but the large differences between A and B vaccine groups are in favor of the short course vaccination and challenge. Viewing the stats in Fig. 2C, 2D and 2E strengthen conclusion of efficacy. Clinical evaluations, using weight loss, is strong component for clinical outcome. Were necropsies done? A table of the findings in supplementary data would add weight to the clinical conclusions drawn.*

Reply: Necropsies was done on a few but not all euthanized animals due to COVID-19. Results of necropsies, therefore, are not available for complete clinical evaluation.

2) *Comment: Line 151 The main points of the rest of the paper refer to CMI responses, and rightly so since p27 antibody is not known to play a role in control. But, what were the anti-gag IgG antibody responses of the other two groups. Was group B also positive? Group C should be negative Fig. S2 only shows group A*.

Reply: Additional tests for anti-gag IgG antibody responses have been performed with macaque samples from Group B. As expected, and mentioned by our reviewers, the anti-gag IgG antibody responses was induced in Group B. Fig. S2 has been updated accordingly.

3) *Comment: In the interest of clarity, the macaque numbers given can cause issues with record keeping and data identification. Are these monkey numbers the house numbers from Foshan University Dept. of Veterinary Medicine? If not, can the animal house numbers be used?*

Reply: The animal house numbers of the macaques were their original assigned numbers: Group A: 150705, 150706, 150707, 150708 Group B: 150701, 160306, 160307; Group C: 150702, 160308, 160309, 171001, 171002, 171003, 171011, 171012, 180505. We have included both the original animal facility no. and the Foshan animal facility numbers (Table S4 and manuscript lines 439-440)

4) *Comment: Outbred Ch Rh lines 182 and 400 – Were outbred Ch Rh purpose bred or wild-caught? This point may seem trivial, but could be important for follow-up studies to reproduce results with larger numbers and move the program forward to clinical studies.*

Reply: Our experimental animals were not wild-caught. They were purchased from a breeding colony based in Guangdong province with birth records. The manuscript is revised accordingly (line 437-439).

**Part II – Major Issues: Key Experiments Required for Acceptance**

Reviewer 1

No comments from Reviewer 1.

Reviewer 2

1) *Comment: Changes will improve the clarity of the MS and data presentation, but are discretionary. Only point 4 is mandatory because it will help to protect the authors for verification of these preclinical studies. Wild caught and purpose bred animals are very different because of the environments and their immune systems can vary.*

Reply: We fully agree with the comments. All our macaques were purchased from the same breeding colony based in Guangdong province with birth records for record keeping. The manuscript is revised accordingly (line 437-439).

**Part III – Minor Issues: Editorial and Data Presentation Modifications**

Reviewer 1

1) *Comment: Can author speculate if there are any quality or quality difference in T cell generated from other already established T cell-based Vaccine strategy in NHP?*

Reply: Brief discussion on the T cell responses induced by various T cell vaccines has been added to lines 365-375 as follow. “Our PD1-based vaccine induced CD8 T cell response against a similar breadth of T cell epitopes as to the heterologous rAd26 prime rAd5 boost vaccine regimen^16, 18^, but was narrower than the RhCMV-based vaccine^34, 44^. Nevertheless, the PD1-based vaccine achieved prolonged viral suppression in all vaccinated macaques. Importantly, our epitope mapping analysis reveals that the PD1-based vaccine successfully induces functional T cell responses that are responsive towards SHIV_SF162P3CN_ infection in the out-bred macaques examined. This result demonstrates the direct involvement of vaccine-elicited T cells during viral challenge. Compared to the heterologous vaccinia prime and adenovirus boost vaccination strategy that we examined previously^12^, the homologous PD1-based DNA vaccination has the potential to prime a stronger anti-viral T cell response, providing an optimized vaccination schedule is followed.”.

2) *Comments: Three effector memory CD8 identified in scRNAseq is interesting, but it is unclear if this is unique from T cell induced by natural infection or other T cell-based Vaccination strategy.*

Reply: A brief discussion about GNLY expression on T cells has been added to lines 394-396 as follow. “Enhanced expression of GNLY in CD8^+^ T cells has been reported in humans vaccinated with a live attenuated shingles vaccine and in antigen-specific T cells in humans vaccinated with yellow fever vaccination^53, 54^.” We also uploaded the single-cell RNA sequencing data to NCBI Gene Expression Omnibus (GEO) and quoted the accession number (GSE171783) in the manuscript for data access (line 552-554).

Reviewer 2

1) *Comment: There are some syntax/grammatical issues here and there for example Line 402 – Welfares and conditions of all animals…should be, Animal welfare and condition of all animals…*

Reply: We have revised the sentence in line 435.

2) *Comment: Line 113 Chinese Rhesus are a sub-species of rhesus macaque group, not a different species. Recommend for line 111 …first passaged the pathogenic SHIVSF162P3 strain isolated from an Indian-origin rhesus macaque with simian AIDS in peripheral blood mononuclear cell (PBMC) of Chinese–origin rhesus macaques to avoid* *immune response to cellular antigens of these 2 different sub-species of Rhesus monkeys…*

Reply: We have revised the sentence in line 131-132.

3) *Comment: Fig. 2B color coding is difficult to follow. Change groups A and B to different colors to make comparisons easier to follow.*

Reply: We have revised the color-coding as suggested.

4) *Comment: Line 56 add reference number 14.*

Reply: We add the reference as suggested in line 75.

5) *Comment: Line 404 … Animals were euthanized to avoid unnecessary suffering when reached humane endpoint, change to…when the experimental endpoint was reached. Humane treatment is guaranteed by approval from Foshan University and need not be repeated in this context.*

Reply: We have revised the sentence in line 436 as suggested.
